# Blood transcriptome analysis of patients with uncomplicated bacterial infection and sepsis

**DOI:** 10.1186/s13104-021-05488-w

**Published:** 2021-02-27

**Authors:** Velma Herwanto, Benjamin Tang, Ya Wang, Maryam Shojaei, Marek Nalos, Amith Shetty, Kevin Lai, Anthony S. McLean, Klaus Schughart

**Affiliations:** 1 Department of Intensive Care Medicine, Nepean Hospital, Sydney, Australia; 2 Centre for Immunology and Allergy Research, The Westmead Institute for Medical Research, Sydney, Australia; 3 Faculty of Medicine, Universitas Tarumanagara, Jakarta, Indonesia; 4 Centre for Infectious Diseases and Microbiology, the Westmead Institute for Medical Research, Sydney, Australia; 5 Department of Emergency Medicine, Westmead Hospital, Sydney, Australia; 6 Department of Infection Genetics, Helmholtz Centre for Infection Research, Braunschweig, Germany; 7 University of Veterinary Medicine Hannover, Hannover, Germany; 8 Department of Microbiology, Immunology and Biochemistry, University of Tennessee Health Science Center, Memphis, TN USA

**Keywords:** Sepsis, Uncomplicated infection, Whole blood transcriptome

## Abstract

**Objectives:**

Hospitalized patients who presented within the last 24 h with a bacterial infection were recruited. Participants were assigned into sepsis and uncomplicated infection groups. In addition, healthy volunteers were recruited as controls. RNA was prepared from whole blood, depleted from beta-globin mRNA and sequenced. This dataset represents a highly valuable resource to better understand the biology of sepsis and to identify biomarkers for severe sepsis in humans.

**Data description:**

The data presented here consists of raw and processed transcriptome data obtained by next generation RNA sequencing from 105 peripheral blood samples from patients with uncomplicated infections, patients who developed sepsis, septic shock patients, and healthy controls. It is provided as raw sequenced reads and as normalized log_2_ transformed relative expression levels. This data will allow performing detailed analyses of gene expression changes between uncomplicated infections and sepsis patients, such as identification of differentially expressed genes, co-regulated modules as well as pathway activation studies.

## Objective

Sepsis is one of the most significant disease burdens in the world [1]. A better understanding of its disease mechanism is urgently needed to facilitate development of new therapy for sepsis. Several putative disease mechanisms have been suggested, including endothelial dysfunction, coagulation dysregulation and abnormal immune response [2]. Among these, abnormal immune response is thought to play the most critical role [3]. The abnormal immune response in sepsis is characterized by impaired innate and adaptive immune responses; both of which have been shown to strongly correlate with poor patient outcomes (*e.g.* increased secondary infection and reduced survival) [4, 5]. Recent studies aimed to determine pathways that are associated with impaired cellular metabolism in the immune cells of sepsis patients [6, 7]. These studies have identified several defective cellular pathways (*e.g.* inhibited mitochondrial complex activity and oxygen consumption, reduced ATP production) across different sepsis populations [8, 9] However, these studies share a common limitation–they were conducted in patients with established sepsis or in the late stage of sepsis. As a result, it is uncertain whether impaired cellular metabolism are also present in the immune cells of infected patients prior to the development of sepsis. Here, we report raw and processed transcriptome data obtained by next generation RNA sequencing from peripheral blood samples of patients with uncomplicated infections, patients who developed sepsis and septic shock patients.

## Data description

Patients with infection were recruited from the emergency department. Subjects were eligible if they aged 18 years or older; presented within the last 24 h with an infection, defined as either (1) positive pathogen identification in any body fluids sampled for microbiological culture, or (2) a suspicion of infection (as determined by the treating physician) and received antibiotics. Exclusion criteria: (1) decision not to actively treat or resuscitate the patient at admission; and (2) inability to consent the patient. The study participants were assigned into sepsis and uncomplicated infection groups, based on their Sequential Organ Failure Assessment (SOFA) score on admission (≥ 2 vs. < 2), in accordance with the international consensus definition of sepsis (“Sepsis-3”)[10]. Septic shock subjects were recruited from the department of intensive care medicine of Nepean Hospital, New South Wales, Australia from December 2017 to February 2019. Recruited septic shock subjects had to fulfil the sepsis criteria as above with persisting hypotension requiring a vasopressor to maintain mean arterial pressure ≥ 65 mmHg and having a serum lactate level > 2 mmol/L despite adequate volume resuscitation as defined in Sepsis-3 [10]. Healthy volunteers, aged 18 or older, from different age groups were recruited at the Westmead Institute for Medical Research and in Magdeburg, Germany. Healthy subjects with recent (within prior 14 days) infection/under antimicrobial medication and subjects under immunosuppressive drugs were not included in the study.

Whole blood was collected on admission and another blood sample was collected 3–5 days later for follow up (FU samples). It was collected in PAXgene Blood RNA tubes and RNA was isolated using PAXgene Blood RNA Kit. Globin mRNA was depleted from total RNA and a strand-specific RNA sequencing library was generated. The library was sequenced with an average of 40 million reads per RNA sample. Reads were quality checked, then trimmed. Trimmed reads were mapped to the human genome and mapped reads were counted. Residual reads to beta-globin were set to 1000. Raw counts were then normalized and log_2_ transformed using DESeq2 (version 1.16.1, [11]). Sex was validated by gene expression for Y-specific genes and corrected if different from recorded sex.

Data are provided as raw sequence data and as normalized log_2_ transformed relative expression values. In addition, we provide a quality control report, a summary statistics for the patients, a detailed sample description for each patient with age, sex, and infection severity (40 Healthy = Hlty, 12 uncomplicated infections = Inf1_P, 20 sepsis = Seps_P, 19 septic shock = Shock_P, 4 follow-up sepsis = Seps_FU, 10 follow-up septic shock = Shock_FU), and a detailed material and methods description.

Our dataset represents a highly valuable resource to study the biology of sepsis and to evaluate biomarkers for severe sepsis in humans which may allow prognoses of development of severe disease. It will allow to perform more detailed analyses of the gene expression changes between uncomplicated infections and sepsis patients, such as identification of differentially expressed genes, co-regulated modules as well as pathway activation studies. Furthermore, our data provides a valuable resource to replicate and validate findings from other studies (Table 1).

**Table 1:** Overview of data files/data sets

Label	Name of data file/data set	File types (file extension)	Data repository and identifier (DOI or accession number)
Data file 1	Raw sequence files	Fastq	Sequence Read Archive https://identifiers.org/ncbi/insdc.sra:SRP273118
Data file 2	Normalized expression values	Text (txt)	Gene Expression Omnibus (https://identifiers.org/geo:GSE154918)
Data set 3	ST1_QC_Sepsis_080221.xlsx Quality control of raw reads before trimming, after trimming, uniquely mapped reads and percentage of mapped reads	Excel	Figshare https://doi.org/10.6084/m9.figshare.13740400.v1
Data set 4	ST2_summary_table_080221.xlsx Summary statistics for patient groups	Excel	Figshare https://doi.org/10.6084/m9.figshare.13740400.v1
Data set 5	ST3_Patients_characteristics_080221.xlsx Description of patients characteristics	Excel	Figshare https://doi.org/10.6084/m9.figshare.13740400.v1
Data set 6	S4_Material_Methods_080221.docx Detailed Material and Methods	Word	Figshare https://doi.org/10.6084/m9.figshare.13740400.v1

## Limitations

Our study has some limitations. The patients were recruited from different settings, emergency department and intensive care units. The sequence of pathophysiology might be different since the intensive care unit patients are recruited later after the onset of the disease. Some unavailable follow-up data in each group of infection limit the power of analysis.

## Data Availability

Raw transcriptome data are available in Sequence Read Archive: https://identifiers.org/ncbi/insdc.sra:SRP273118 [12]. Processed transcriptome data are available in Gene Expression Omnibus: https://identifiers.org/geo:GSE154918 [13]. The quality control report, a detailed sample description for each patient with age, sex, and sepsis status, and detailed description of Material and Methods are available in the figshare repository: 10.6084/m9.figshare.13740400.v1 [14].
